# CD8+ T Cell Control of HIV—A Known Unknown

**DOI:** 10.1371/journal.ppat.1000728

**Published:** 2010-01-29

**Authors:** Miles P. Davenport, Janka Petravic

**Affiliations:** Complex Systems in Biology Group, Centre for Vascular Research, University of New South Wales, Sydney, New South Wales, Australia; NIH/NIAID, United States of America

The former US Secretary of Defense, Donald Rumsfeld, once famously divided our areas of understanding into three categories: “known knowns … things we know we know”, “known unknowns … [things we] know we … do not know”, and “unknown unknowns—the ones we don't know we don't know” [1]. In HIV immunity, the role of CD8+ cytotoxic T lymphocytes (CTLs) in controlling virus has been thought to be “known” for over two decades. The recognition of viral peptides by CD8+ T cells was initially demonstrated by showing that CTLs could lyse chromium-labeled target cells presenting viral epitopes [2]. Multiple lines of evidence have subsequently supported the important role for CTLs in controlling HIV replication [3],[4]. However, most of these studies have not explicitly examined the mechanisms by which viral control is achieved. Because of the name (“cytotoxic”) and the early assays for detection (measuring cytolysis using chromium release in vitro), it is usually assumed that CD8+ T cells control HIV infection by killing HIV-infected cells. Indeed, the use of the terms CD8+ T lymphocyte and cytotoxic T lymphocyte have become almost interchangeable in HIV. Thus, the mechanism of CTL control of infection through the cytolysis of infected cells has fallen into the category of a “known known”.

Two papers in this issue of *PLoS Pathogens* present a major challenge to the assumption that CD8+ T cells kill HIV-infected cells [5],[6]. Using very similar methodology, Klatt et al. and Wong et al. used an approach of depleting CD8+ T cells during simian immunodeficiency virus (SIV) infection of macaques to study the mechanisms of CD8+ T cell control of virus. What makes their studies different from previous studies of CD8+ T cell depletion is that they also treated animals with anti-retroviral therapy (ART) after CD8+ T cell depletion. Because ART blocks subsequent rounds of viral infection, it has been used extensively over the last decade to study the turnover of HIV-infected cells [7],[8]. Combining CD8 depletion and ART treatment allowed the two groups to study the death rate of SIV-infected cells in the presence and absence of CD8+ T cells. Remarkably, the groups found no difference in the decay rate of virus in CD8-depleted compared to control animals. This indicates that CD8+ T cells had no significant impact on the death rate of infected cells, suggesting that a high death rate is an intrinsic property of infected cells. Since CD8+ T cells have a clear role in viral control, they must act via some mechanism other than lysis of infected cells.

On the surface, we could simply accept that CD8+ T cell control of HIV infection acts via non-cytolytic mechanisms such as the production of cytokines and/or chemokines, and make that our new credo. Such a shift in beliefs would be supported by the demonstration that the ability of CD8+ T cells to produce multiple cytokines is associated with good viral control in HIV, suggesting an important role for cytokines [9],[10]. However, it is difficult to reconcile the results of the Klatt and Wong papers with other recent in vivo and in vitro results (see Table 1). First amongst these are studies of CD8+ T cell depletion in acute SIV infection [3],[11]. These studies demonstrate that in the absence of CD8+ T cells, the virus level rises to its “normal” peak in acute infection, but then does not decline from the peak for a prolonged period. The simplest interpretation of this result is that, in the absence of CD8+ T cells, the peak number of infected cells does not decline, but remains constant because these cells are not dying. However, two other observations make this unlikely: First, the increased loss of CD4+ T cells in CD8-depleted animals indicates that many cells are dying. Second, the Klatt and Wong results show that infected cells die at the same rate independent of the presence of CD8+ T cells in chronic infection, so why would things be so different in primary infection? How can viral load stay so high in CD8-depleted animals in acute infection if infected cells are dying at the same rate? One explanation would be that CD8+ T cell depletion leads to activation of CD4+ T cells; since SIV preferentially infects activated cells, the infected CD4+ T cells are dying, but these dying cells are being replaced because of the high level of CD4+ T cell activation. However, a recent study of CD8+ T cell depletion in acute SIV infection has excluded this possibility, by showing that CD4+ T cell activation is neither necessary nor sufficient to produce the prolonged high viral loads observed in vivo [11].

**Table 1:** Evidence for and against Cytolytic Control of HIV.

		Experiment	Result	Inference
In vivo	Non-cytolytic control	CD8 depletion in chronic SIV infection [15]	Increase in viral load is too rapid to be accounted for by reduced infected cell death.	Effect of CD8+ T cells is to inhibit viral production. Rapid rise in viral load due to dis-inhibition of production.
		CD8 depletion + ART in chronic SIV infection [5],[6]	Decay rate of viral load is the same in presence and absence of CD8+ T cells.	CD8+ T cells do not affect death rate of productively infected cells.
		CD8+ T cell-inducing vaccine in SHIV infection [16],[17]	The rate of decay of viral load after the peak of infection doesn't change even when peak is reduced by 10- to 100-fold.	CD8+ T cell control of SHIV in vaccinated animals does not alter death rate of productively infected cells.
	Cytolytic control	In vivo injection of peptide-pulsed cells in SIV [18]	Rapid killing of cells bearing SIV peptides	High peptide dose and absence of HIV immune avoidance mechanisms make results difficult to interpret.
		CD8 depletion in acute SIV infection [3],[11]	Viral load follows normal pattern of primary infection until the peak viral load, then stays elevated.	CD8+ T cells kill SIV-infected cells, causing decline in viral load after the peak. In the absence of CD8+ T cell killing, SIV-infected CD4+ T cells are long-lived, so that viral load does not decay.
In vitro		In vitro killing of peptide-pulsed/vaccinia-infected cells by CD8+ T cells [2]	Rapid killing in chromium release assays	High peptide dose and absence of HIV immune avoidance mechanisms make results difficult to interpret.
		Culture of SIV-infected cells with virus-specific CD8+ T cells	Inhibition of viral growth by CD8+ T cell supernatant [19],[20],[21]	Inhibition of viral growth does not require cell contact.
			Lysis of HIV-infected cells [12]	CD8+ T cells require in vitro activation prior to assay.

A non-cytolytic mechanism of CD8+ T cell control in vivo is also difficult to reconcile with in vitro results directly demonstrating CD8+ T cell lysis [2],[12]. Early studies of in vitro lysis relied on pulsing target cells with HIV peptides, or infecting cells with vaccinia virus bearing HIV proteins [2]. These experiments may not reflect the normal levels of peptide-MHC present on HIV-infected cells, both because they may involve unphysiologically high doses of peptide, and because HIV Nef is known to down-regulate MHC class I expression in infected cells. However, recent studies have demonstrated the recognition and lysis of HIV-infected primary target cells in vitro, presumably bearing physiological levels of peptide, and suffering any effects of Nef and other proteins on antigen presentation [12]. Notably, these studies required prior activation of the CD8+ T cells to demonstrate high levels of killing, but still suggest a role for direct killing of infected cells. A compromise between the cytolytic and non-cytolytic camps is the idea that infected cells may be killed in a “window period” before they start to produce virus [13],[14]. However, this still fails to explain all of the experimental observations.

The Klatt and Wong papers provide a direct challenge to the assumption that CD8+ T cell cytolysis of infected cells is the mechanism of viral control in HIV. However, it is difficult to reconcile the combination of in vivo and in vitro results investigating CD8+ T cell activity in HIV. Given the enormous effort invested in the development of HIV vaccines specifically directed at inducing CD8+ T cell responses, it is somewhat disconcerting to admit that CD8+ T cell function in HIV remains a “known unknown”.
